# Entry point of machine learning in axial spondyloarthritis

**DOI:** 10.1136/rmdopen-2023-003832

**Published:** 2024-02-15

**Authors:** Yuening Chen, Hongxiao Liu, Qing Yu, Xinning Qu, Tiantian Sun

**Affiliations:** Department of Rheumatology, China Academy of Chinese Medical Sciences Guang'anmen Hospital, Beijing, China

**Keywords:** Spondylitis, Ankylosing, Machine Learning, Autoimmune Diseases

## Abstract

Axial spondyloarthritis (axSpA) is a globally prevalent and challenging autoimmune disease. Characterised by insidious onset and slow progression, the absence of specific clinical manifestations and biomarkers often leads to misdiagnosis, thereby complicating early detection and diagnosis of axSpA. Furthermore, the high heterogeneity of axSpA, its complex pathogenesis and the lack of specific drugs means that traditional classification standards and treatment guidelines struggle to meet the demands of personalised treatment. Recently, machine learning (ML) has seen rapid advancements in the medical field. By integrating large-scale data with diverse algorithms and using multidimensional data, such as patient medical records, laboratory examinations, radiological data, drug usage and molecular biology information, ML can be modelled based on real-world clinical issues. This enables the diagnosis, stratification, therapeutic efficacy prediction and prognostic evaluation of axSpA, positioning it as an emerging research topic. This study explored the application and progression of ML in the diagnosis and therapy of axSpA from five perspectives: early diagnosis, stratification, disease monitoring, drug efficacy evaluation and comorbidity prediction. This study aimed to provide a novel direction for exploring rational diagnostic and therapeutic strategies for axSpA.

**WHAT IS ALREADY KNOWN ON THIS TOPIC**

- Machine learning has rapidly advanced in medicine, emerging as a central focus of research across disciplines. However, a comprehensive review of machine learning (ML) applications in axial spondyloarthritis is currently lacking.

**WHAT THIS STUDY ADDS**

- This manuscript outlines ML applications and advancements for axial spondyloarthritis diagnosis and treatment exploring five key areas: early diagnosis, stratification, disease monitoring, drug efficacy assessment and comorbidity prediction.

**HOW THIS STUDY MIGHT AFFECT RESEARCH, PRACTICE OR POLICY**

- Aimed at inspiring researchers in rheumatology and machine learning, this study anticipates heightened focus on this interdisciplinary domain, fostering specialisation and cutting-edge development.

## Introduction

Axial spondyloarthritis (axSpA) falls under the classification of SpA. Depending on the presence of radiographic changes in sacroiliitis, it is further categorised into non-radiographic axSpA (nr-axSpA) and ankylosing spondylitis (AS), with the latter also referred to as radiographic axSpA.^1^ axSpA is a globally prevalent, intractable autoimmune disease primarily characterised by chronic progressive inflammation of the sacroiliac joint and spinal rigidity. It predominantly affects the brain and males under the age of 30 years, who are at their physical peak, significantly impacting patients’ quality of life and leading to a high disability rate. The insidious onset and slow progression of axSpA, coupled with the absence of specific clinical manifestations and biomarkers, often results in misdiagnosis, thereby complicating early detection and diagnosis. Furthermore, the high heterogeneity of axSpA, its complex pathogenesis and the lack of specific drugs pose challenges for traditional classification standards and treatment guidelines in meeting the demands of personalised treatment.

**Figure 1:**
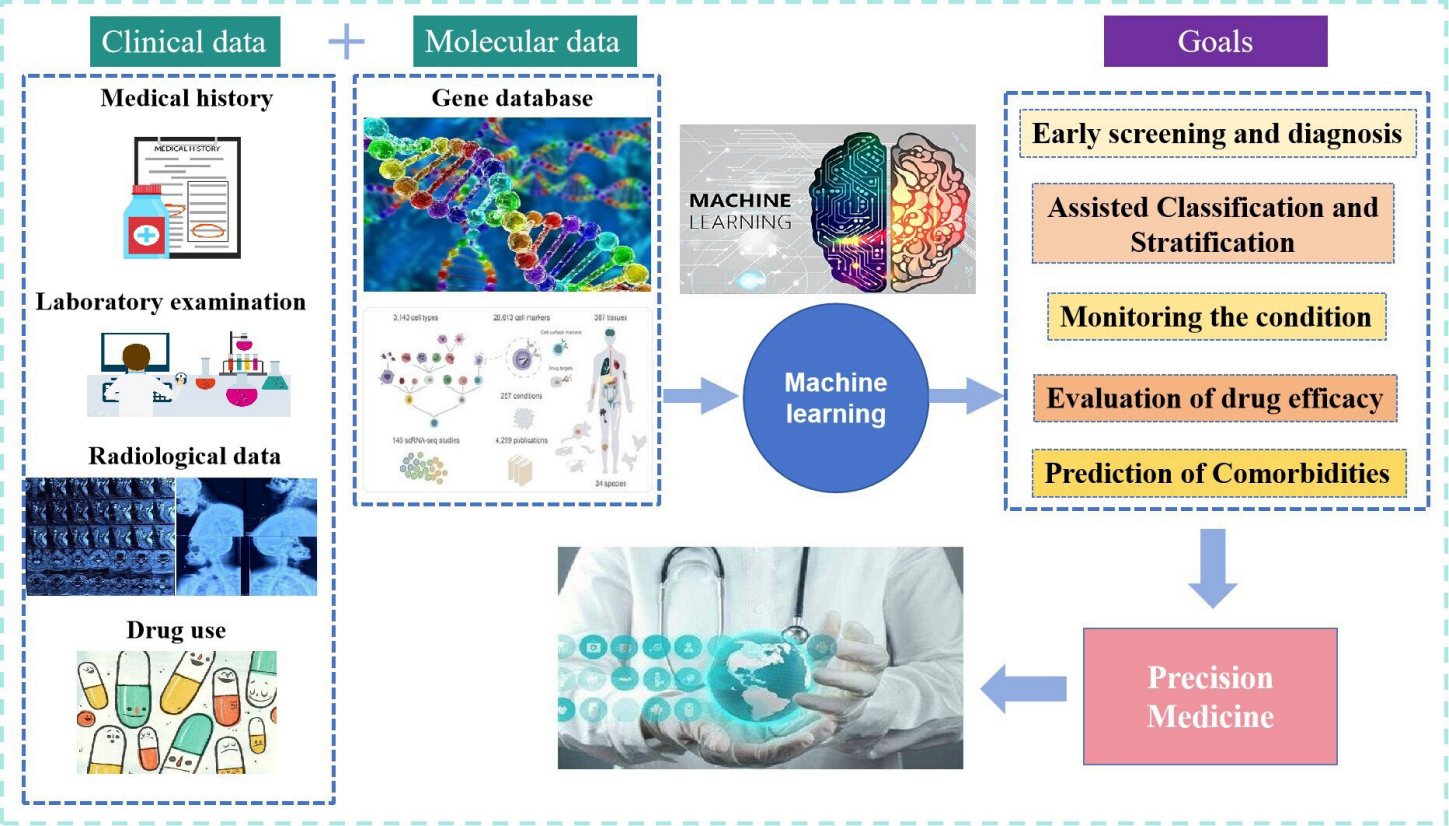
Machine learning for precision medicine in axSpA. This study aims to use machine learning techniques with patient clinical and molecular data to facilitate early diagnosis, stratification, disease monitoring, drug efficacy evaluation and comorbidity prediction in axSpA, ultimately achieving precision medicine. All photos are sourced from Baidu. axSpA, axial spondyloarthritis.

In recent years, machine learning (ML) has rapidly advanced within the medical domain. By integrating large-scale data with diverse algorithms and using multidimensional data, such as patient medical records, laboratory examinations, radiological data, drug usage and molecular biology information, ML can be modelled based on real-world clinical issues. This enables the diagnosis, classification, prediction of therapeutic efficacy and prognostic evaluation of axSpA, marking it as an emerging research hotspot. In 2020, the EULAR proposed that with the accumulation of large-scale clinical and omics data, computational methods and statistical models beyond human capabilities should be employed to fully harness the potential of ML technology, thereby providing reasonable expectations for clinical diagnosis and treatment.^2^ Building on this, the present study delineates the application and progression of ML in the diagnostic and therapeutic processes of axSpA across five key areas: early diagnosis, stratification, disease monitoring, drug efficacy evaluation and comorbidity prediction. This study aimed to offer a novel direction for the exploration of more rational diagnostic and therapeutic strategies for axSpA (see table 1 and figure 1).

**Table 1:** Current status of published articles on machine learning in axSpA

Goals	Year of publication	Author	Journal	Input data	Machine learning technology	Predictive effectiveness
Precision medicine						
Early screening and diagnosis	2020^7^	Deodhar *et al*	*Clin Rheumatol*	Medical claims database medical records	Optimise the linear regression model A/B	Model A/B PPV: 6.24% Simplified linear regression model PPV: 2.55%
	2022^9^	Zhu *et al*	*Rheumatol Ther*	Laboratory indicators	LASSO, SVM-RFE, RF and Nomograms	Training cohort AUC: 0.87 Validation cohort AUC: 0.82
	2022^10^	Ye *et al*	*Rheumatology*	MRI and medical records	mRMR, LASSO, multivariable logistic regression analysis and Nomograms	Rad-score：training/validation cohort AUC: 0.82. The clinical-radiomics nomogram model: training/validation cohort AUC: 0.9
	2022^11^	Wen *et al*	*Front Genet*	GEO database	LASSO, SVM-RFE, RF and Nomograms	AUC>0.84
	2022^12^	Han *et al*	*Front Immunol*	GEO database	WGCNA, SVM-RFE	AUC>0.7
	2020^8^	Zhao *et al*	*Rheumatology*	Electronic health records	Logistic regression, LASSO penalised and multimodal automated phenotyping	AUC: 0.93
Assisted classification and stratification	2023^14^	Zhang *et al*	*Journal of Digital Imaging*	CT images	3D convolutional neural network	Validation set AUC: 0.91, 0.80, 0.96 Test set AUC: 0.94, 0.82, 0.93
	2020^15^	Castro-Zunti *et al*	*Comput Med Imaging Graph*	CT and age	RF, K-NN	AUC>0.9
	2022^16^	Bressem *et al*	*Radiology*	MRI	Deep learning	Inflammatory changes: AUC: 0.94 Structural changes: AUC: 0.89
	2022^17^	Lin *et al*	*Rheumatology*	MRI	Attention U-net	AUC: 0.92
	2020^18^	Tenório *et al*	*Int J Comput Assist Radiol Surg*	MRI-based radiomics biomarkers	Mann-Whitney U	AUC>0.8
Monitoring the condition	2023^20^	Baek *et al*	*Arthritis Res Ther*	mSASSS and clinical data	Artificial neural network, generalised linear model	ANN RMSE: 2.83 GLM RMSE: 2.99
	2019^21^	Gossec *et al*	*Arthritis Care Res (Hoboken)*	Wearable activity trackers	Multiclass Bayesian	Positive predictive value 91%
Evaluate the efficacy of the drug	2020^22^	Lee *et al*	*Sci Rep*	The baseline demographic and laboratory data	ANN,logistic regression, support vector machine, random forest and XGBoost models	ANN model AUC: 0.783, logistic regression, support vector machine, random forest and XGBoost models (AUC: 0.719, 0.699, 0.761 and 0.713)
	2022^23^	Wang *et al*	*JAMA Netw Open*	The baseline demographic, laboratory data and medication history	Logistic regression, linear discriminant analysis, SVM, GBM and RF	AUC>0.7
	2021^24^	Barata *et al*	*JMIR Med Inform*	Electronic medical records	Joint models	/
Prediction of comorbidities	2022^27^	Zhang *et al*	*Front Genet*	GEO database	LASSO, RF, XGBoost and SVM	SVM: AS AUC 0.7, LBMD AUC: 0.76
	2020^29^	Navarini *et al*	*Rheumatol Ther*	Clinical data	SVM, RF and KNN	AUC values for the ML algorithms were: 0.70 for SVM, 0.73 for RF and 0.64 for KNN

ANN, artificial neural network; AUC, area under curve; 3D, three-dimensional; GBM, gradient boosting machine; GEO, Gene Expression Omnibus; GLM, generalised linear model; K-NN, K-nearest neighbour; LBMD, low bone density; PPV, positive predictive value; RF, random forest; RMSE, root mean square error; SVM-RFE, support vector machine recursive feature elimination; WGCNA, Weighted Gene Co-expression Network Analysis; XGBoost, extreme gradient boosting.

### Overview of ML

ML is a scientific discipline that enables computers to learn and perform tasks similar to humans. It self-learns and trains by using sample data, uncovering patterns, establishing standards and accomplishing specific objectives. The essence of ML lies in the interplay between large datasets and diverse algorithms.^3^ Depending on the presence of labels, ML is categorised into supervised, semisupervised and unsupervised learning, which are commonly used statistical learning methods within the medical domain. Common algorithms include K-nearest neighbours (KNN), decision trees, random forest (RF), gradient boosting machine (GBM), support vector machine (SVM) and convolutional neural networks (CNN). The primary tasks of these algorithms are classification and regression and discovering the mapping relationship between input variables and output data, which are frequently employed to predict disease onset risk and enhance diagnostic efficiency. Deep learning (DL), a subset of ML, is used within ML for data representation learning. It is based on multilayer neural networks (input, middle and output layers), thereby facilitating deep data mining and enhancing image and speech recognition, visual object recognition and natural language processing.^4^ In recent years,^5^ DL image processing technology has become increasingly sophisticated, with fully automatic medical image detection, classification and segmentation emerging as research hotspots. Among these, CNN, a type of feedforward neural network, is one of the representative DL algorithms extensively applied in medical imaging processing. A CNN comprises a data input layer, convolutional layer, pooling layer and fully connected layer, among other network structures. Its core lies within the convolutional layer. Unlike traditional ML algorithms that require preliminary feature extraction from images, the CNN convolutional layer can directly use all information contained within the image, significantly enhancing accuracy and efficiency. In summary, ML can leverage multidimensional data, such as text and images, and through computational methods and statistical models beyond human capabilities, it can provide reasonable expectations for clinical diagnosis and treatment.

### Entry points of ML in axSpA

#### Early screening and diagnosis of axSpA with ML

AS poses diagnostic challenges due to the absence of specific and clinical manifestations, such as lower back pain, which often overlap with other diseases, leading to delayed diagnosis. Research indicates that it takes approximately 14 years to correctly diagnose AS.^6^ The application of ML enhances the reliability and accuracy of disease diagnosis systems. Deodhar *et al*^7^ used a medical claims database to optimise a linear regression model for predicting AS. Model A was constructed to predict AS diagnosis during the first period from 2006 to 2015, and model B was employed to validate patients diagnosed with AS during the first period from 2015 to 2018. By retrospectively studying the diagnosis of AS at different time points, early diagnosis was achieved with a positive predictive value (PPV) of 6.24%, which was superior to that of the simplified linear regression model (PPV: 2.55%). Zhao *et al*^8^ employed real-world electronic health records, integrated natural language processing with International Classification of Disease (ICD) codes, optimised logistic regression, Least absolute shrinkage and selection operator(LASSO) regression and multimodal automatic phenotypes through three different algorithms to correctly identify axSpA. Their approach achieved a specificity of 94%, a sensitivity of 78% and an area under curve (AUC) of 0.93, all superior to using ICD alone (AUC: 0.8–0.87). Zhu *et al*^9^ applied LASSO regression, SVM recursive feature elimination (SVM-RFE) and RF to screen the feature indicators of routine blood tests, liver function and kidney function in 384 AS cases and 360 normal individuals. They constructed a model using line plots of the screened erythrocyte sedimentation rate (ESR), red cell count, mean platelet volume, albumin, aspartate aminotransferase and creatinine. The AUC in the validation queue was 0.82, marking the first attempt to establish a diagnostic model for AS using laboratory indicators.

Researchers have integrated clinical and radiological data for the joint diagnosis of axSpA. Ye *et al*^10^ selected 638 patients diagnosed with axSpA (n=424) and non-axSpA (n=214) randomly dividing them into training (n=447) and validation cohorts (n=191). They combined clinical risk factors with sacroiliac MRI radiomics and employed minimum redundancy, maximum relevance (absolute) and LASSO regression to construct an optimal radiomics feature model. The Radiomics Score (Rad-score) in the training and validation cohorts was 0.82. Furthermore, the integration of the Rad-score with six clinical risk prediction factors: age, sex, disease course, ESR, HLA-B27 and C reactive protein (CRP) was conducted. Through multivariate logistic regression analysis, a clinical factor-radiomics model was constructed, achieving an AUC of 0.9 in both the training and validation cohorts. This suggests that the integration of clinical radiomics can enhance clinical diagnostic value.

ML can use gene expression subtypes as labels to generate algorithms for scoring disease characteristics. Wen *et al*^11^ employed the high-throughput Gene Expression Omnibus (GEO) database and applied LASSO regression, SVM-RFE and RF to select hub genes differentially expressed between AS and healthy individuals. Validation across different gene datasets yielded AUC values exceeding 0.84. IL2RB and ZDHHC18 were identified as potential diagnostic markers for AS in peripheral blood, facilitating early diagnosis of AS. Han *et al*^12^ used the GEO database and applied Weighted Gene Co-expression Network Analysis and SVM-RFE to select differential genes with AUC values exceeding 0.7. Peripheral blood was collected from 40 patients with AS and healthy individuals to validate differential mRNA expression. Among these, *CXCR6*, *IL17RA* and *LRRFIP1* were positively correlated with the disease activity score Bath ankylosing spondylitis disease activity index (BASDAI) and could serve as potential biomarkers. In summary, ML can assist in early AS diagnosis by leveraging patient medical records, laboratory indicators, radiological data and molecular and biological information.

#### ML-assisted classification and stratification of axSpA

Radiological examination plays a crucial role in the diagnosis and stratification of SpA. Through radiography, CT and MRI, distinctions can be made between axSpAs, nr-axSpAs and pSpAs. Concurrently, SpA staging can be facilitated by assessing inflammation or structural damage in the sacroiliac joint and spine. ML is becoming increasingly popular in image feature recognition and is being extensively employed in medical image processing. CT is essential for aiding the diagnosis of AS, with specific advantages in detecting structural changes.^13^ Zhang *et al*^14^ used the no-new-UNet to segment sacroiliac joint CT images and employed a three-dimensional (3D) CNN based on the grading results of three senior radiologists to reclassify sacroiliac arthritis using a three-classification method. Grades 0–1 were designated as class 1, grade 2 as class 2 and grades 3–4 as class 3. The AUC exceeded 0.8, outperforming the radiologists. Castro-Zunti *et al*^15^ applied a grey-level co-occurrence matrix and local binary pattern to optimise KNN and RF as ML algorithms. They used CT texture information and AS age as input features to predict sacroiliac joint erosion in young and older patients. The AUC values were 0.97 and 0.91, respectively, with the prediction performance surpassing that of radiologists with 9 years of experience.

Bressem *et al*^16^ conducted a retrospective multicentre study, collecting data from 593 patients across 5 centres for MRI analysis. They developed a DL tool based on AS sacroiliac joint MRI images, scoring for inflammatory changes (AUC: 0.94) and structural changes (AUC: 0.89), achieving a differential diagnosis of AS grading from the imaging level. In addition, Lin *et al*^17^ also identified sacroiliac joint MRI bone marrow oedema to distinguish whether AS is in the active phase (AUC: 0.92) through an attention medical segmentation DL model. Tenório *et al*^18^ collected 47 sacroiliac joint MRIs, 37 of which were diagnosed as SpA (27 axSpA and 10 pSpA). They used ML for MRI radiomics analysis and screening for biomarkers based on image texture. This showed certain advantages in diagnosing AS and distinguishing between axSpA and pSpA (AUC>0.8). Existing research uses various ML models to process different radiological images of SpA to achieve disease classification and stratification.

#### ML monitoring of axSpA condition

Approximately 20%–50% of patients with AS exhibit radiographic progression of the spine after 2 years.^19^ ML can effectively predict structural damage to the AS spine. Baek *et al*^20^ collected a total of 555 patients with AS, choosing those with at least 1 year of X-ray follow-up. They combined the mSASSS score from the follow-up X-ray with clinical baseline characteristics and the use of Nonsteroidal Anti inflammatory Drugs(NSAIDs) and tumour necrosis factor-α (TNF-α). A total of 2034 clinical information points from the follow-up period were used to predict mSASSS progression using both generalised linear models (GLMs) and artificial neural network (ANN) models. The ANN model had a root mean squared error of 2.83, outperforming the GLMs 2.99. It effectively captured the complex interactions between variables and their contributions to the mSASSS assessment over time in the fitted model. Gossec *et al*^21^ conducted a longitudinal study over 3 months, using wearable trackers to monitor the physical activity of patients with AS. Patient-reported disease status was evaluated weekly, and a Bayesian network was used to predict AS recurrence, with an average positive prediction rate of 91%. Employing ML to monitor the AS disease status has the potential to interrupt spinal structural damage and predict disease recurrence, thereby delaying disease progression.

#### ML evaluation of drug efficacy in axSpA

Currently, the treatment landscape for axSpA has transitioned to the era of biological agents, and ML plays a pivotal role in predicting the timing of drug intervention and drug efficacy. Lee *et al*^22^ compared ANN, logistic regression, SVM, RF and XGBoost models. They found that, in predicting whether AS should use TNF-α inhibitor (TNFi) early (diagnosed with AS within 6 months early), the predictive performance of the ANN model was superior to that of other traditional models (AUC: 0.783). Simultaneously, it was found that CRP and ESR are the most important baseline features for predicting early TNFi users. Wang *et al*^23^ conducted a retrospective cohort study, collecting data from 10 randomised controlled studies of tumour TNFi versus placebo or antirheumatic drug treatment for active AS, all receiving at least 12 weeks of TNFi treatment. Various demographic characteristics (age, sex), body mass index, laboratory tests (HLA-B27, CRP), disease characteristics (disease course, BASDAI, Bath ankylosing spondylitis functional index(BASFI), patient global assessment(PGA), 36-item short-form(SF-36), uveitis, inflammatory bowel disease, and/or psoriasis history, joint tenderness, and swelling count), medication history and other 27 clinical factors were used as potential predictive factors, and the improvement in ankylosing spondylitis disease activity score(ASDAS) score was used as the outcome indicator. LASSO regression, linear discriminant analysis, SVM, GBM and RF were used as algorithm optimisation models. The study found that CRP, BASDAI, BASFI and age were the most important predictive factors for evaluating the efficacy of TNF. Barata *et al*^24^ proposed a survival analysis method based on multiple models to jointly predict the failure of biological treatment. This method extracts demographic characteristics, comorbidities, medication and other clinical information of patients with SpA electronic medical records. The ASAS 20 was used as the outcome indicator, and Kaplan-Meier, COX regression, and linear mixed model were applied jointly; CRP, ESR, BASDAI and ASDAS were considered the most important predictive factors for the first failure of biological treatment. This method serves as a valuable tool for predicting AS treatment response, offering tailored diagnosis and treatment plans based on the patient’s disease characteristics.

#### ML prediction of comorbidities in axSpA

Patients with axSpA often contend with comorbidities that significantly impact their health and quality of life. ML plays a crucial role in predicting the risk of these comorbidities, thereby contributing to overall patient health improvement. The skeleton is a primary target organ in AS. Inflammation can lead to bone loss and erosion in the vertebrae, resulting in conditions such as low bone density (LBMD), osteoporosis and vertebral fractures in patients with AS. Osteoporosis and vertebral ossification are commonly recognised complications of AS.^25^ Research points out that the incidence of vertebral fractures in patients with AS is approximately 10%, of which approximately 81% are at the cervical level, leading to nerve damage, with an instantaneous death risk of 5.3%–11.3%.^26^ Zhang *et al*^27^ obtained AS and LBMD datasets through the GEO database, used the intersecting genes of the two to establish diagnostic models using LASSO, RF, XGBoost and SVM algorithms and conducted immune infiltration analysis using CIBERSORT. Finally, five genes, *TNF, CCL3, PIK3CG, PTGER2 and IFNAR1*, could be used as potential biomarkers for predicting the risk of LBMD in patients with AS.

Comorbid cardiovascular disease is prevalent in patients with axSpA. Compared with the general population, the mortality rate from cardiovascular disease in patients with AS increases by 20%–40%. The EULAR against rheumatism recommends an annual cardiovascular risk assessment for patients with AS.^28^ Navarini *et al*^29^ conducted a prospective cohort study using the occurrence of a first cardiovascular event in patients with AS as an outcome indicator. Predictive clinical included sex, age, disease activity, blood pressure, blood lipids, CRP, ESR, creatinine levels, presence or absence of atrial fibrillation, and radiological results are predictive factors. Applying SVM (AUC: 0.7), RF (AUC: 0.73) and KNN (AUC: 0.64), they identified CRP levels as the most crucial factor in predicting cardiovascular diseases in patients with AS. This predictive modelling can aid in assessing the risk of disease occurrence and mitigating the occurrence of comorbidities in AS.

### Conclusions

axSpA often manifests with a concealed onset and slow progression, and the absence of specific clinical manifestations and biomarkers often leads to misdiagnosis, posing challenges to its diagnosis and treatment. Consequently, the field of axSpA faces numerous challenges in terms of diagnosis, treatment and prognosis. ML offers a promising avenue for addressing these challenges by being applicable to early diagnosis, stratification, disease monitoring, drug efficacy evaluation and comorbidity prediction of axSpA. Despite its advantages in clinical applications, there are some limitations, such as the practicality of clinical diagnosis and treatment, the need for improved interpretability and transparency of output conclusions, proper handling of patient privacy protection and the absence of high-quality large datasets. To address these challenges, we propose expanding the scale of the dataset by incorporating diverse populations, including different races, genders, ages and other populations, into the model to improve its accuracy. Simultaneously, improvements in data management, output systems and the strengthening of patient privacy protection are essential.

Furthermore, there are many areas to explore in the application of ML in axSpA. Clinical information, laboratory indicators, radiological examinations and multiomics results can be integrated to build multimodal diagnostic models. Advances in proteomics, single-cell sequencing, metabolomics, microbiomics and other systems biology research can further contribute to developing axSpA prediction models at the microscopic molecular level. Exploring the effects of IL-17 and JAK inhibitors on axSpA in terms of drug efficacy is a promising avenue for precision treatment. In addition, as axSpA targets spinal bone structural damage, 3D facial image recognition could be used to explore whether there are differences in facial bone structure.

In summary, despite existing problems and deficiencies in the application of ML in axSpA, ongoing improvements in the accuracy of the developed models allow ML to enhance the overall understanding of axSpA by learning from high-quality big data. It can optimise patient stratification, refine treatment strategies and predict drug efficacy and prognosis, offering innovative approaches for exploring more rational diagnostic and treatment strategies for axSpA. It can play a crucial role in the precision diagnosis and treatment of axSpA, contributing significantly to the advancement of the field.

## References

[1] Sieper J, Poddubnyy D. Axial spondyloarthritis. Lancet 2017;390:73–84. 10.1016/S0140-6736(16)31591-4 28110981

[2] Gossec L, Kedra J, Servy H, et al. EULAR points to consider for the use of big data in rheumatic and musculoskeletal diseases. Ann Rheum Dis 2020;79:69–76. 10.1136/annrheumdis-2019-215694 31229952

[3] Pandit A, Radstake TRDJ. Machine learning in rheumatology approaches the clinic. Nat Rev Rheumatol 2020;16:69–70. 10.1038/s41584-019-0361-0 31908355

[4] Choi RY, Coyner AS, Kalpathy-Cramer J, et al. Introduction to machine learning, neural networks, and deep learning. Transl Vis Sci Technol 2020;9:14. 10.1167/tvst.9.2.14 PMC7347027 32704420

[5] Bizopoulos P, Koutsouris D. Deep learning in cardiology. IEEE Rev Biomed Eng 2019;12:168–93. 10.1109/RBME.2018.2885714 30530339

[6] Feldtkeller E, Khan MA, van der Heijde D, et al. Age at disease onset and diagnosis delay in HLA-B27 negative vs. positive patients with ankylosing spondylitis. Rheumatol Int 2003;23:61–6. 10.1007/s00296-002-0237-4 12634937

[7] Deodhar A, Rozycki M, Garges C, et al. Use of machine learning techniques in the development and refinement of a predictive model for early diagnosis of ankylosing spondylitis. Clin Rheumatol 2020;39:975–82. 10.1007/s10067-019-04553-x 31044386

[8] Zhao SS, Hong C, Cai T, et al. Incorporating natural language processing to improve classification of axial spondyloarthritis using electronic health records. Rheumatology (Oxford) 2020;59:1059–65. 10.1093/rheumatology/kez375 31535693 PMC7850056

[9] Zhu J, Lu Q, Liang T, et al. Development and validation of a machine learning-based nomogram for prediction of ankylosing spondylitis. Rheumatol Ther 2022;9:1377–97. 10.1007/s40744-022-00481-6 35932360 PMC9510083

[10] Ye L, Miao S, Xiao Q, et al. A predictive clinical-radiomics nomogram for diagnosing of axial spondyloarthritis using MRI and clinical risk factors. Rheumatology (Oxford) 2022;61:1440–7. 10.1093/rheumatology/keab542 34247247

[11] Wen J, Wan L, Dong X. Novel peripheral blood diagnostic biomarkers screened by machine learning algorithms in ankylosing spondylitis. Front Genet 2022;13:1032010. 10.3389/fgene.2022.1032010 36386830 PMC9663919

[12] Han Y, Zhou Y, Li H, et al. Identification of diagnostic mRNA biomarkers in whole blood for ankylosing spondylitis using WGCNA and machine learning feature selection. Front Immunol 2022;13:956027. 10.3389/fimmu.2022.956027 36172367 PMC9510835

[13] de Koning A, de Bruin F, van den Berg R, et al. Low-dose CT detects more progression of bone formation in comparison to conventional radiography in patients with ankylosing spondylitis: results from the SIAS cohort. Ann Rheum Dis 2018;77:293–9. 10.1136/annrheumdis-2017-211989 29127092

[14] Zhang K, Luo G, Li W, et al. Automatic image segmentation and grading diagnosis of sacroiliitis associated with AS using a deep convolutional neural network on CT images. J Digit Imaging 2023;36:2025–34. 10.1007/s10278-023-00858-1 37268841 PMC10501961

[15] Castro-Zunti R, Park EH, Choi Y, et al. Early detection of ankylosing spondylitis using texture features and statistical machine learning, and deep learning, with some patient age analysis. Comput Med Imaging Graph 2020;82:101718. 10.1016/j.compmedimag.2020.101718 32464565

[16] Bressem KK, Adams LC, Proft F, et al. Deep learning detects changes indicative of axial spondyloarthritis at MRI of sacroiliac joints. Radiology 2023;307:e239007. 10.1148/radiol.239007 37093751

[17] Lin KYY, Peng C, Lee KH, et al. Deep learning algorithms for magnetic resonance imaging of inflammatory sacroiliitis in axial spondyloarthritis. Rheumatology (Oxford) 2022;61:4198–206. 10.1093/rheumatology/keac059 35104321

[18] Tenório APM, Faleiros MC, Junior JRF, et al. A study of MRI-based radiomics biomarkers for sacroiliitis and spondyloarthritis. Int J Comput Assist Radiol Surg 2020;15:1737–48. 10.1007/s11548-020-02219-7 32607695

[19] Poddubnyy D, Haibel H, Listing J, et al. Baseline radiographic damage, elevated acute-phase reactant levels, and cigarette smoking status predict spinal radiographic progression in early axial spondylarthritis. Arthritis Rheum 2012;64:1388–98. 10.1002/art.33465 22127957

[20] Baek I-W, Jung SM, Park Y-J, et al. Quantitative prediction of radiographic progression in patients with axial spondyloarthritis using neural network model in a real-world setting. Arthritis Res Ther 2023;25:65. 10.1186/s13075-023-03050-6 37081563 PMC10116698

[21] Gossec L, Guyard F, Leroy D, et al. Detection of flares by decrease in physical activity, collected using wearable activity trackers in rheumatoid arthritis or axial spondyloarthritis: an application of machine learning analyses in rheumatology. Arthritis Care Res (Hoboken) 2019;71:1336–43. 10.1002/acr.23768 30242992

[22] Lee S, Eun Y, Kim H, et al. Machine learning to predict early TNF inhibitor users in patients with ankylosing spondylitis. Sci Rep 2020;10. 10.1038/s41598-020-75352-7 PMC7679386 33219239

[23] Wang R, Dasgupta A, Ward MM. Predicting probability of response to tumor necrosis factor inhibitors for individual patients with ankylosing spondylitis. JAMA Netw Open 2022;5:e222312. 10.1001/jamanetworkopen.2022.2312 35289857 PMC8924712

[24] Barata C, Rodrigues AM, Canhão H, et al. Predicting biologic therapy outcome of patients with spondyloarthritis: joint models for longitudinal and survival analysis. JMIR Med Inform 2021;9:e26823. 10.2196/26823 34328435 PMC8367135

[25] Sambrook PN, Geusens P. The epidemiology of osteoporosis and fractures in ankylosing spondylitis. Ther Adv Musculoskelet Dis 2012;4:287–92. 10.1177/1759720X12441276 22859927 PMC3403252

[26] Huang J, Bai H, Tan Q, et al. Instantaneous death risk, conditional survival and optimal surgery timing in cervical fracture patients with ankylosing spondylitis: a national multicentre retrospective study. Front Immunol 2022;13:971947. 10.3389/fimmu.2022.971947 36189242 PMC9521542

[27] Zhang D, Liu J, Gao B, et al. Immune mechanism of low bone mineral density caused by ankylosing spondylitis based on bioinformatics and machine learning. Front Genet 2022;13:1054035. 10.3389/fgene.2022.1054035 36468006 PMC9716034

[28] Peters MJL, Symmons DPM, McCarey D, et al. EULAR evidence-based recommendations for cardiovascular risk management in patients with rheumatoid arthritis and other forms of inflammatory arthritis. Ann Rheum Dis 2010;69:325–31. 10.1136/ard.2009.113696 19773290

[29] Navarini L, Caso F, Costa L, et al. Cardiovascular Risk Prediction in Ankylosing Spondylitis: From Traditional Scores to Machine Learning Assessment. Rheumatol Ther 2020;7:867–82. 10.1007/s40744-020-00233-4 32939675 PMC7695785

